# Identification and functional annotation of metabolism‐associated lncRNAs and their related protein‐coding genes in gastric cancer

**DOI:** 10.1002/mgg3.427

**Published:** 2018-07-10

**Authors:** Xiaoyan Mo, Tianwen Li, Yi Xie, Linwen Zhu, Bingxiu Xiao, Qi Liao, Junming Guo

**Affiliations:** ^1^ Department of Biochemistry and Molecular Biology Zhejiang Key Laboratory of Pathophysiology Medical School of Ningbo University Ningbo China; ^2^ Department of Preventative Medicine Zhejiang Key Laboratory of Pathophysiology Medical School of Ningbo University Ningbo China

**Keywords:** coexpression network, long noncoding RNA, metabolism, NDUFB6, TOPORS‐AS1

## Abstract

**Background:**

Long noncoding RNAs (lncRNAs) play important roles in carcinogenesis. However, the roles of metabolism‐associated lncRNAs in cancers are still unclear.

**Methods:**

A microarray of metabolism‐associated lncRNAs was used to detect their expression patterns between gastric cancer and paired nontumorous tissues. Its results and gastric cancer differential gene expression data from public databases were used to screen the metabolic pathway‐associated lncRNAs. A metabolic network with microRNAs (miRNAs), lncRNAs, and protein‐coding genes was further constructed. Finally, the expression of TOPORS antisense RNA 1 (TOPORS‐AS1), a screened highly expressed lncRNA and its associated protein‐coding gene, NADH: ubiquinone oxidoreductase subunit B6 (NDUFB6), were verified by reverse transcription polymerase chain reaction.

**Results:**

A total of eight upregulated and one downregulated lncRNAs and 25 upregulated and 20 downregulated protein‐coding genes were found to be involved in metabolism in gastric cancer. Within the lncRNAs–miRNAs–mRNAs metabolic network, 78 miRNA‐target links, 546 positive coexpression relationships, and 191 protein–protein interactions were found. The expression of TOPORS‐AS1 and its associated gene, NDUFB6 in gastric cancer tissues was significantly lower than that in adjacent nontumor tissues. Moreover, NDUFB6 expression was associated with the invasion and distal metastasis of gastric cancer.

**Conclusions:**

The metabolism‐associated lncRNAs play important roles in the occurrence of gastric cancer.

## 1 BACKGROUND

Gastric cancer, one of the most common cancers worldwide, is a major public health problem (Wang et al., 2018). The proportion of gastric cancer incidence varies regionally. South America, Eastern Europe, and especially eastern Asia are the most susceptible regions (Yari et al., 2016). Globally, most gastric cancer patients are unresectable when diagnosed (Chen, Li, Zhao, Xiao, & Guo, 2017). The prognosis for advanced gastric cancer is still poor (Li et al., 2017). Therefore, exploring the molecular mechanisms underlying the occurrence and development of gastric cancer are urgently needed.

Metabolism is broadly defined as the sum of biochemical processes in living organisms that either produce or consume energy (DeBerardinis & Thompson, 2012). Altered metabolism, a hallmark of cancers, contributes to cancers from initiation, growth, and maintenance to malignant transformation (Hanahan & Weinberg, 2011). Identifying these metabolic alterations and the underlying regulatory mechanisms in cancers could open a window of opportunity for therapeutic intervention.

Most cancer cells prefer to produce energy by glycolysis rather than oxidative phosphorylation via the tricarboxylic acid cycle, even under aerobic conditions (Warburg effect) (Hanahan & Weinberg, 2011). Several studies have showed that the expression of glucose transporter‐1 (GLUT‐1), which is involved in the glycolytic pathway, is significantly correlated with prognosis of gastric carcinoma (Kawamura et al., 2001; Noguchi et al., 1999). Additionally, GLUT1 is significantly associated with the clinical stages and depth of invasion of patients with gastric cancer (Yan, Wang, Chen, Li, & Fan, 2015). Metastasis‐associated in colon cancer‐1 (MACC1), a long noncoding RNA (lncRNA), contributes to the Warburg effect by upregulating the expression and activities of a series of glycolytic enzymes in gastric cancer cells, including hexokinase, pyruvate dehydrogenase kinase, and lactate dehydrogenase (Lin et al., 2015). Amino acid metabolism in gastric cancer cells is altered as well. For example, phosphoserine, serine, cysteine, tyrosine, isoleucine, glutamine, and valine are increased in gastric cancer specimens compared to adjacent normal mucosa (Wu et al., 2010). Moreover, it is reported that valine has revealed the greatest fold change in gastric cancer patients compared to controls (Song et al., 2012).

lncRNAs are usually described as transcripts longer than 200 nucleotides with no open reading frame (Shao et al., 2016). According to the distance from protein‐coding genes, lncRNAs can be categorized into one or more types, including sense, antisense, bidirectional, intronic, and intergenic lncRNAs (Li, Mo, Fu, Xiao, & Guo, 2016). Current knowledge regarding the functions of lncRNAs has indicated that they can influence cellular proliferation, cell cycle regulation, survival, apoptosis, metastasis, or immune response (Hashad, Elbanna, Ibrahim, & Khedr, 2016; Sun, Yang, Xu, Xie, & Guo, 2016). Furthermore, several lncRNAs regulate metabolism, including glycometabolism and lipid metabolism. For instance, lincRNA p21 can interact with hypoxia‐inducible factor‐1α (HIF‐1α) and then modulate glycolysis (Yang, Zhang, Mei, & Wu, 2014). Through the mTOR–STAT3⁄miR‐143 pathway, urothelial cancer‐associated 1 (UCA1) promotes glycolysis by upregulating hexokinase 2 (HK2), the first pivotal enzyme in the glycolytic pathway (Li, Li, Wu, Xue, & Chen, 2014). Moreover, highly upregulated in liver cancer (HULC) regulates abnormal lipid metabolism through the miR‐9–mediated RXRA signaling pathway (Cui et al., 2015).

Since there is no research addressing the associations between the expression profiles of lncRNAs and metabolism in gastric cancer, we performed a human metabolism pathway lncRNA microarray analysis to characterize the expression profiles of lncRNAs and their potential protein‐coding genes that are critical in the metabolic pathway. A series of bioinformatics prediction methods were also applied for the functional annotation of the differentially expressed lncRNAs. More importantly, the typical lncRNA and its associated metabolic gene were confirmed by experiments in gastric cancer tissues.

## 2 MATERIALS AND METHODS

### 2.1 Ethical compliance

All aspects of this study were approved by the Human Research Ethics Committee of Ningbo University (IRB No. 20120303). Written informed consent was obtained from all patients.

### 2.2 Patient selection

Samples of gastric cancer tissues and corresponding adjacent nontumorous tissues were obtained from surgical or biopsy specimens from August 2011 to February 2014 at Yinzhou People's Hospital, China. Tissue samples were directly preserved in RNA fixer (Bioteke, Beijing, China) after removal from the body and stored at −80°C until total RNA isolation. The adjacent nontumorous tissues were 5 cm from the edge of the tumor, and there were no obvious tumor cells, as confirmed by two pathologists. There was no chemotherapy, radiotherapy, targeted therapy, or other therapies prior to the upper gastrointestinal endoscopy examination or operation. Tumors were staged according to the tumor node metastasis (TNM) staging system of the International Union Against Cancer (5th edition). Histological grade was assessed following the National Comprehensive Cancer Network clinical practice guideline of oncology (NCCN Guidelines V.1.2011).

### 2.3 Microarray analysis

Three paired specimens were obtained from patients with poorly and moderately differentiated gastric cancer. The LncPath™ human metabolism pathway lncRNA microarray (Arraystar, Rockville, MD, USA) was used, which contains probes for both lncRNAs and mRNAs to simultaneously profile the expression of 965 lncRNAs and 458 protein‐coding gene targets related to the metabolic signaling pathway. In this microarray, the lncRNAs whose genes are located at or near the protein‐coding genes are critical in the metabolic pathway, and the lncRNAs that have high possibilities of being competing endogenous RNAs (ceRNAs) of the key metabolic pathway genes were carefully collected from authoritative databases using rigorous selection processes.

For microarray analysis, the Agilent Array platform (Agilent Technologies, Santa Clara, CA, USA) was employed. Sample preparation and microarray hybridization were performed based on the manufacturer's standard protocols. Briefly, total RNA from each sample was amplified and transcribed into fluorescent cRNA with using Arraystar Flash RNA Labeling protocol (Arraystar). The labeled cRNAs were hybridized onto the LncPath™ human metabolism array (6 × 7K, Arraystar).

GenePix Pro 6.0 (Axon) was used to analyze scanned images for grid alignment and data extraction. Quantile normalization and subsequent data processing were performed using R. After quantile normalization of the raw data, lncRNAs and mRNAs for which at least three of the six samples exhibited expression were retained for further analyses. Statistically significant differentially expressed lncRNAs between the two groups were identified through volcano plot filtering. Differentially expressed lncRNAs between two samples were identified through fold‐change filtering. Hierarchical clustering was performed to determine the distinguishable lncRNA expression patterns among samples. The normalized intensity for each lncRNA and mRNA was presented as a log^2^‐transformed pattern; fold changes >1.5 and *P *<* *0.05 were selected as significantly differentially expressed.

### 2.4 Functional annotation of LncRNAs in the gastric cancer network

First, we downloaded lncRNA protein‐coding gene coexpression relationships calculated from gastric‐associated expression profiles from the Co‐LncRNA database (Zhao et al., 2015). For each lncRNA in the coexpression network, the enriched Gene Ontology (GO) biological process (BP) of its direct coexpressed protein‐coding genes was calculated by using a hypergeometric test with an adjusted *P*‐value less than 0.05 and assigned for the lncRNAs. Then, as there is only one coexpression gastric network in the Co‐LncRNA database (Zhao et al., 2015) and the number of lncRNAs involved is limited, we downloaded all lncRNA protein‐coding gene coexpression relationships in the Co‐LncRNA database (Zhao et al., 2015) and constructed a coexpression network with the links detected from at least 50 datasets. Finally, we performed the functional annotation for each differently expressed lncRNA using the same method as above.

### 2.5 Coexpression network construction

All protein‐coding genes and lncRNAs on our microarray dataset were used to calculate the Pearson correlation coefficient (PCC) with one another. Then, the p‐values of the PCCs were estimated by Fisher's asymptotic method using the WGCNA package in R (Langfelder & Horvath, 2008) and were further adjusted by FDR using the multitest package in R. Finally, the coexpressed pairs were selected if the adjusted *P*‐values were less than 0.05.

### 2.6 Quantitative RT‐PCR

Real‐time quantitative reverse transcription–polymerase chain reaction (qRT‐PCR) is the gold standard for data confirmation. To examine the results of the LncPath™ human metabolism pathway lncRNA microarray, cDNA was generated from 2.0 μg of total RNA using the GoScript Reverse Transcription (RT) System (Promega, Madison, WI, USA). PCR was performed using the GoTaq qPCR Master Mix (Promega) on an Mx3005P Real‐Time PCR system (Stratagene, La Jolla, CA, USA). The sequences of the PCR primers were as follows: 5′‐ACCCACTCCTCCACCTTTGAC‐3′ (forward) and 5′‐TGTTGCTGTAGCCAAATTCGTT‐3′ (reverse) for glyceraldehyde‐3‐phosphate dehydrogenase (GAPDH); 5′‐CTTCAGCACCCAGAAACTCCAA‐3′ (forward) and 5′‐GCAAGCAGCAAGTAAGAAGCG‐3′ (reverse) for TOPORS antisense RNA 1 (TOPORS‐AS1; ENSG00000235453); 5′‐TCCATGGGGTATACAAAAAGAG‐3′ (forward) and 5′‐GGAAATTCTTTCATTGGTGGA‐3′ (reverse) for NADH: ubiquinone oxidoreductase subunit B6 (NDUFB6; ENSG00000165264; OMIM: 603322). The thermal cycling conditions were as follows: 95°C at 10 min for a hot start and then 40 cycles at 95°C for 15 s, 58°C for 30 s, and 72°C for 30 s. The relative expression levels of detected genes were calculated using the comparative threshold cycle method, and the relative fold change in gene expression was calculated by using the following formula: 2^−ΔΔ*C*q ^= 2^−[Δ*C*q (tumor sample) − Δ*C*q (normal control)]^, where Δ*C*_q _= *C*_q_ (detected gene)* − C*_q_ (GAPDH). All results were expressed as the mean ± SD of three independent experiments (Shao et al., 2016).

### 2.7 Statistical analysis

All experimental data were analyzed using SPSS version 22.0 (SPSS Inc., Chicago, IL, USA). Differences in expression levels between gastric cancer tissues and adjacent nontumor tissues were analyzed using a *t* test. The correlation between TOPORS‐AS1 or NDUFB6 levels and clinicopathological factors of patients with gastric cancer was analyzed by one‐way analysis of variance (ANOVA). *P *<* *0.05 was considered as statistically significant.

## 3 RESULTS

### 3.1 Identification of metabolic LncRNAs and protein‐coding genes in gastric cancer

**Figure 1:**
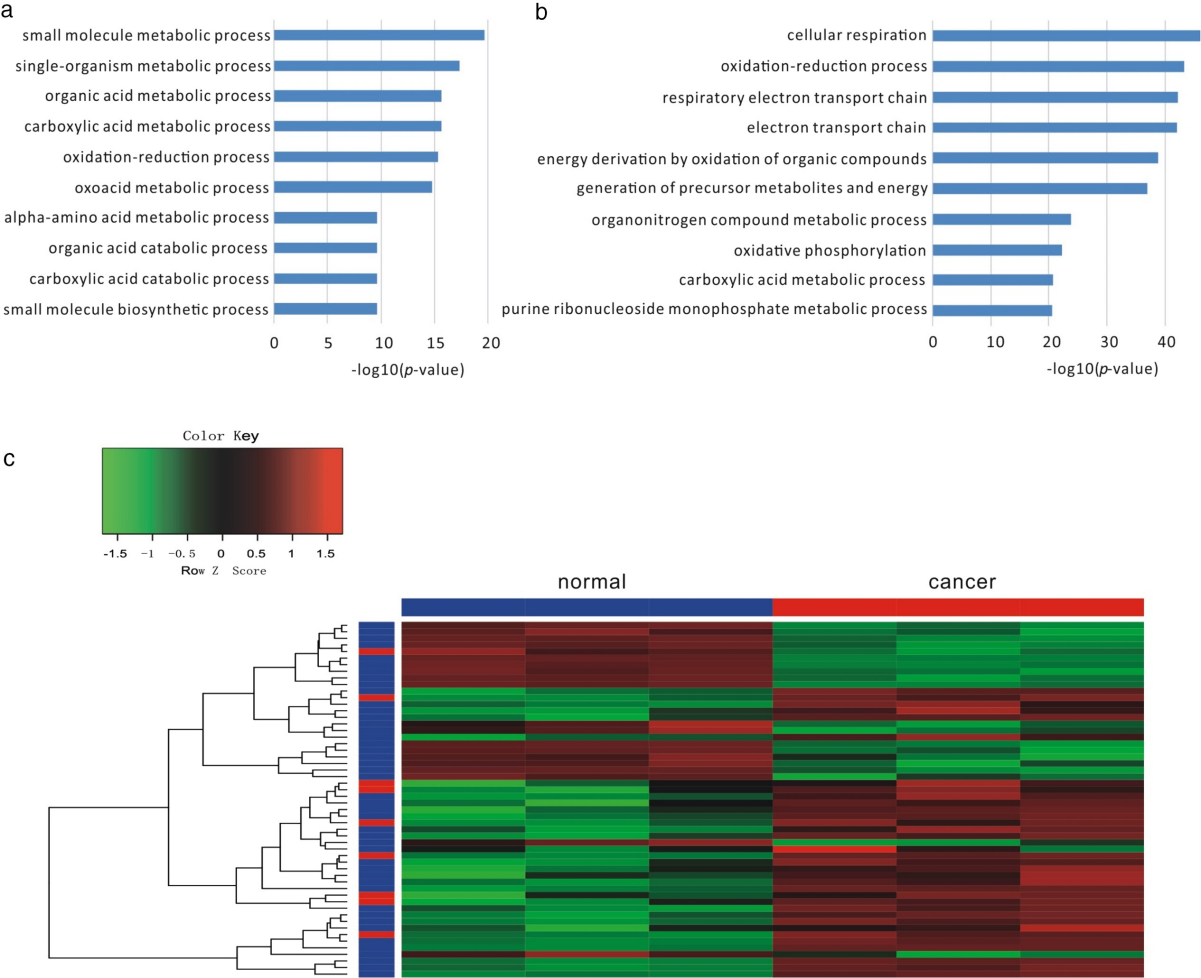
The top 10 enriched GO terms of differently expressed genes. (a) The top 10 enriched GO terms of upregulated genes. (b) The top 10 enriched GO terms of downregulated genes. (c) Heatmap of differentially expressed genes in our metabolic microarray. The blue bar in the row represents protein‐coding genes, while the red bar in the row represents lncRNAs

We applied a human metabolism pathway lncRNA microarray with 965 lncRNAs and 458 protein‐coding gene targets related to the metabolic signaling pathway on three gastric cancer samples from three independent patients and paired normal samples. Differential expression analysis identified 45 upregulated and 73 downregulated protein‐coding genes and 63 upregulated and 51 downregulated lncRNAs (data accessible at NCBI GEO database, accession GSE96856; https://www.ncbi.nlm.nih.gov/geo/query/acc.cgi?acc=GSE96856). The enriched GO BP terms of upregulated protein‐coding genes were mainly small molecule metabolic process, single‐organism metabolic process, organic acid metabolic process, carboxylic acid metabolic process, and oxidation–reduction process and so on (Figure 1a), while for the downregulated protein‐coding genes, the functions are mainly cellular respiration, oxidation–reduction process, respiratory electron transport chain, electron transport chain, and energy derivation by oxidation of organic compounds and so on (Figure 1b).

We further combined the results of differentially expressed genes of gastric cancer from the Cancer RNA‐seq Nexus database, which includes information on differentially expressed lncRNAs and protein‐coding genes in multiple cancer types (Li, Sun, et al., 2016), and recovered the same differentially expressed lncRNAs and protein‐coding genes in the same direction as the metabolism‐associated genes in gastric cancer. In total, we found eight upregulated lncRNAs and 25 upregulated protein‐coding genes, one downregulated lncRNA and 20 downregulated protein‐coding genes that may be involved in metabolism in gastric cancer (Figure**** 1c). Some of them were well studied in gastric cancer or other kinds of cancers. For example, glucose‐6‐phosphate dehydrogenase (G6PD), which participates in the pentose phosphate pathway, was overexpressed in gastric cancer tissues compared to normal stomach mucosa tissues, and its level was associated with tumor size, depth of invasion, lymph node metastasis, distant metastasis, TNM stage, and survival rate (Wang et al., 2012), indicating that G6PD expression level was an independent prognostic factor for gastric cancer patients after radical resection. In addition, lncRNA SLC25A25 antisense RNA 1 (SLC25A25‐AS1) was recently found to be dysregulated and associated with cell proliferation and chemoresistance in colorectal cancer (Li et al., 2016c), suggesting its similar function in gastric cancer.

### 3.2 Functional annotation of LncRNAs based on the LncRNA protein‐coding gene coexpression network

Next, to annotate the functions of unknown differently expressed lncRNAs in gastric cancer, we first obtained the lncRNA protein‐coding gene coexpression network of gastric cancer from the Co‐LncRNA database (Zhao et al., 2015). We applied a hub‐based method using the same procedures as in a previous report (Liao et al., 2011). As a result, three lncRNAs with more than three coexpressed protein‐coding genes were annotated. The roles of some lncRNAs or genes have been reported before. For example, HLA complex P5 (HCP5) was annotated with immune response, defense response, and other immune system‐related GO terms. In a previous study, one genetic variant (rs3099844) of HCP5 was found to contribute to nevirapine‐induced Stevens Johnsons Syndrome/toxic epidermal necrolysis susceptibility in a population from Mozambique (Borgiani et al., 2014), suggesting the immune‐associated function of HCP5. In addition, HCP5 has also been annotated with some metabolic processes such as cellular protein metabolic process and cellular macromolecule metabolic process (adjusted *P *<* *0.01). Another lncRNA TREC was also annotated with metabolic‐related process, and the enriched Kyoto Encyclopedia of Genes and Genomes (KEGG) pathways show that it may be involved in the ribosome and Alzheimer's disease. Recently, it was reported that three polymorphisms (rs12696304, rs3772190, rs16847897) of the telomerase RNA component (*TERC*) gene were associated with Alzheimer's disease (Scarabino, Broggio, Gambina, Pelliccia, & Corbo, 2017). The last lncRNA, DiGeorge syndrome critical region gene 9 (DGCR9), was also annotated with metabolic processes such as phenylpropanoid metabolic process, response to follicle‐stimulating hormone, sodium‐independent organic anion transport, diacylglycerol metabolic process, and cardiac atrium morphogenesis. All these facts suggest the accuracy of functional annotation in our method.

**Figure 2:**
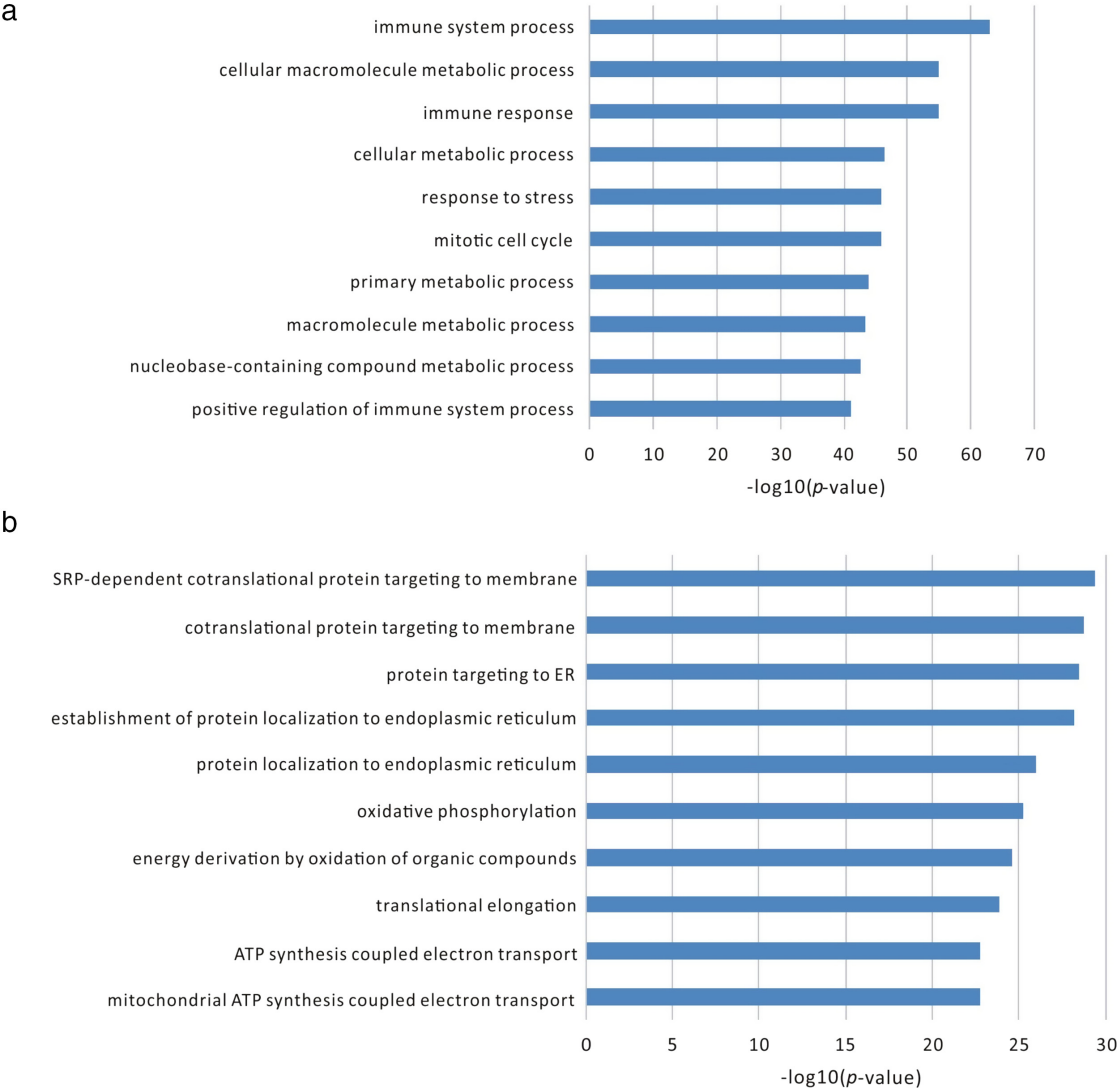
The predicted functions of lncRNAs based on protein‐coding gene‐lncRNA coexpression relationships. (a) The top 10 predicted functions of lncRNA HCP5. (b) The top 10 predicted functions of lncRNA TOPORS‐AS1

To further annotate the functions of metabolic‐associated lncRNAs, we downloaded all 241 datasets of lncRNA protein‐coding gene coexpression relationships in the Co‐LncRNA database (Zhao et al., 2015) and constructed a lncRNA protein‐coding gene coexpression network where the links were detected in at least 50 datasets. In this way, four lncRNAs were annotated with functions. HCP5, the same as mentioned above, was also annotated as immune system process, immune response, response to stress and other metabolic processes such as cellular macromolecule metabolic process and nucleobase‐containing compound metabolic process (Figure**** 2a). Three other lncRNAs, namely, AFAP1 antisense RNA 1 (AFAP1‐AS1), MAFG antisense RNA 1 (MAFG‐AS1), and TOPORS‐AS1, were annotated as metabolic‐related processes. One of them, TOPORS‐AS1, was annotated as cellular respiration, oxidation–reduction process, and translational termination and so on (Figure 2b).

### 3.3 Metabolic network analysis of MiRNA, LncRNAs, and protein‐coding genes in gastric cancer

**Figure 3:**
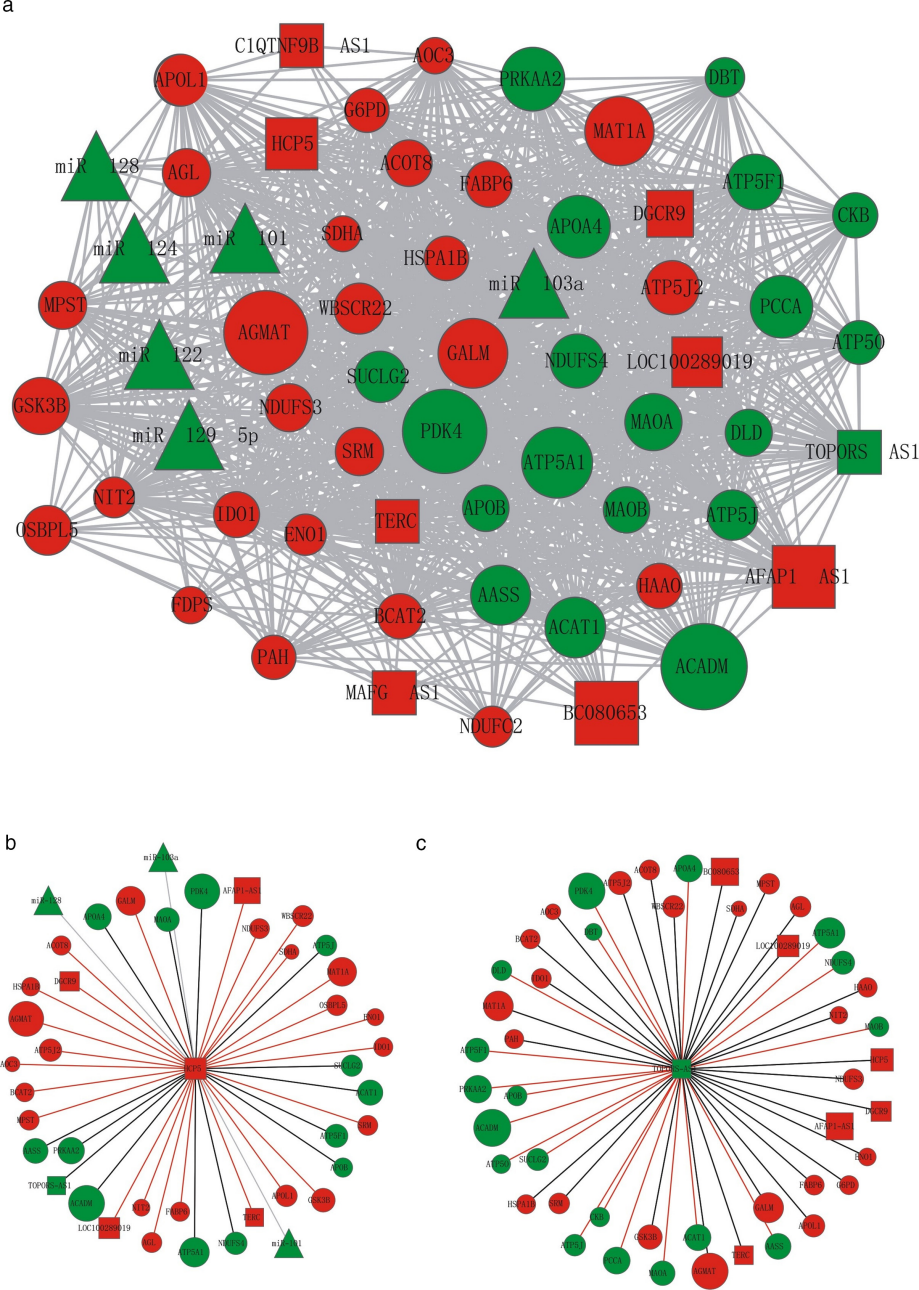
The metabolic network of miRNAs, lncRNAs and protein‐coding genes in gastric cancer. (a) Visualization of the metabolic network of miRNAs, lncRNAs and protein‐coding genes in gastric cancer. Red nodes represent upregulated genes in gastric cancer. Blue nodes represent downregulated genes in gastric cancer. The sizes of nodes represent the absolute log^2^ value of fold change. (b) The subnetwork of lncRNA HCP5. The meanings of node color and size are the same as in (a). The red edges represent positive coexpression links while the black edges represent negative coexpression links. The gray edges represent relationships between miRNAs and lncRNAs. (c) The subnetwork of lncRNA TOPORS‐AS1. The meanings of node color and size are the same as in (a), while the meaning of edge color is the same as in (b)

To find the associations among the differentially expressed protein‐coding genes and lncRNAs, a metabolic network with both lncRNAs and protein‐coding genes was constructed by combining the following relationships: coexpression relationships calculated by our microarray dataset, protein–protein interactions from the STRING database (Szklarczyk et al., 2015), miRNA‐target regulations from the miRcode database (Jeggari, Marks, & Larsson, 2012) with corresponding miRNAs differentially expressed in the opposite direction in gastric cancer according to the miRcancer database (Xie, Ding, Han, & Wu, 2013). Finally, the metabolic network was composed of 78 miRNA‐target links, 546 positive coexpression and 468 negative expression relationships, and 191 protein–protein interactions (PPIs) wherein 152 were the same as the coexpression relationships (71 positive links and 81 negative links; Figure 3a). Among them, five relationships involved miRNA–lncRNAs. For example, lncRNA TERC was identified as associated with miR‐129‐5p while C1QTNF9B‐AS1 was found to be regulated by miR‐103a. The aberrant expression of these lncRNAs in gastric cancer may be caused by several key miRNAs.

In the metabolic network, HCP5 was coexpressed with 34 metabolic‐related protein‐coding genes and five lncRNAs and was regulated by three miRNAs (Figure 3b). The miRNAs were miR‐128, miR‐101, and miR‐103a, which are downregulated in gastric cancer. There have been no reports of regulation between miRNAs and HCP5 until now, but our results suggest that the expression of HCP5 may be regulated by miRNAs in gastric cancer. TOPORS‐AS1 is coexpressed with 42 protein‐coding genes and six lncRNAs (Figure 3c), suggesting the metabolic function of TOPORS‐AS1.

### 3.4 Verification of TOPORS‐AS1 and NDUFB6 downregulation in gastric cancer

**Figure 4:**
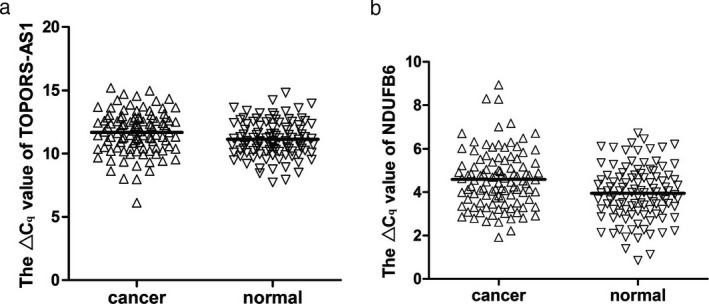
The expression level (Δ*C*_q_) of TOPORS‐AS1 and NDUFB6 mRNA in gastric cancer tissues compared with normal tissues. **(**a) The expression of TOPORS‐AS1 is downregulated in gastric cancer. (b) The expression of NDUFB6 mRNA is downregulated in gastric cancer. Bigger Δ*C*_q_ means higher expression of NDUFB6

We performed qRT‐PCR to verify the co‐expression network of metabolic‐associated lncRNAs and their coexpressed protein‐coding genes. We randomly selected 103 patients with gastric cancer. We found that TOPORS‐AS1 expression was significantly lower in gastric cancer tissues than in adjacent nontumor tissues (*P *<* *0.01, Figure 4a). According to the abovementioned metabolic network, TOPORS‐AS1 was coexpressed with NDUFB6, which is located on the opposite side of TOPORS‐AS1. Then, we observed the expression pattern of NDUFB6 mRNA and found the same trend (*P *<* *0.001, Figure 4b), as expected. These results suggested the regulatory relationship between them. An association analysis between their expression and clinicopathological features indicated that NDUFB6 expression levels were associated with CA19‐9, invasion and distal metastasis (Table**** 1).

**Table 1:** Association of TOPORS‐AS1 and NDUFB6 expression levels (Δ*C*_q_) in cancer tissues with clinicopathological features of patients with gastric cancer

Characteristics	No. of patients (%)	TOPORS‐AS1		NDUFB6	
		Mean ± SD	*P* value	Mean ± SD	*P* value
Age (y)					
≥60	68 (66.02)	11.78 ± 1.57	0.366	4.57 ± 1.47	0.823
<60	35 (33.98)	11.47 ± 1.76		4.63 ± 1.01	
Gender					
Male	75 (72.82)	11.72 ± 1.59	0.648	4.55 ± 1.31	0.593
Female	28 (27.18)	11.55 ± 1.78		4.71 ± 1.39	
Diameter(cm)					
≥5	54 (52.43)	11.80 ± 1.67	0.390	4.71 ± 1.40	0.355
<5	49 (47.57)	11.52 ± 1.60		4.46 ± 1.24	
Hp					
Positive	35 (33.98)	11.56 ± 1.94	0.617	4.45 ± 1.32	0.453
Negative	68 (66.02)	11.73 ± 1.46		4.66 ± 1.34	
CEA					
Positive	96 (93.20)	11.65 ± 1.66	0.663	4.62 ± 1.33	0.439
Negative	7 (6.80)	11.93 ± 1.35		4.21 ± 1.31	
CA19‐9					
Positive	63 (61.17)	11.49 ± 1.57	0.153	4.26 ± 1.17	0.001
Negative	40 (38.83)	11.96 ± 1.71		5.17 ± 1.42	
Differentiation					
Well	9 (8.70)	11.63 ± 1.05	0.942	3.89 ± 1.10	0.058
Moderate	52 (50.49)	11.62 ± 1.66		4.45 ± 1.31	
Poor	42 (40.78)	11.74 ± 1.72		4.92 ± 1.33	
Invasion					
Tis & T1–T3	39 (37.86)	11.88 ± 1.42	0.322	4.15 ± 1.71	0.007
T4	64 (62.14)	11.55 ± 1.75		4.86 ± 1.35	
Lymphatic metastasis					
N0	37 (35.92)	11.79 ± 1.13	0.568	4.55 ± 1.46	0.819
N1‐3	66 (64.08)	11.60 ± 1.86		4.61 ± 1.26	
Distal metastasis					
M0	94 (91.26)	11.61 ± 1.47	0.259	4.48 ± 1.25	0.004
M1	9 (8.74)	12.26 ± 2.89		5.79 ± 1.61	
TNM stage					
0 & Ι & П	42 (40.78)	11.88 ± 1.33	0.278	4.48 ± 1.42	0.481
Ш & ΙV	61 (59.22)	11.52 ± 1.81		4.67 ± 1.27	

## 4 DISCUSSION

It is well known that lncRNAs play an important role in cancers. They can serve as regulators at the transcriptional or posttranscriptional levels. Several lncRNAs, including HOX transcript antisense RNA (HOTAIR) and H19, are involved in cell proliferation, apoptosis, invasion, migration, and metastasis of gastric cancer in the similar manners as oncogenes or tumor suppressor genes (Cui et al., 2015; Hashad et al., 2016; Li et al., 2014; Li, Mo, et al., 2016 Nie et al., 2018; Shao et al., 2016; Sun et al., 2016; Yang et al., 2014).

Since the roles of cancer metabolic‐associated lncRNAs are largely unclear, in this study, we identified differentially expressed protein‐coding genes and lncRNAs with metabolic processes by combining our microarray and public RNA‐seq results (Figure 1). The metabolic functions of several lncRNAs were further verified based on coexpression networks. Then, we constructed a metabolic network of gastric cancer with miRNAs, lncRNAs, and protein‐coding genes, showing the complex regulations relationships among them (Figure**** 3).

Within the abovementioned coexpression network, one lncRNA, TOPORS‐AS1, whose functions were predicted as cellular respiration, oxidation–reduction process, translational termination and so on (Figure**** 2), and the associated NDUFB6, which is located in the same genomic regions with opposite transcript direction, were both verified to be downregulated in gastric cancer samples by RT‐PCR (Figure**** 4). Association analysis with clinicopathological features found that NDUFB6 may be a new biomarker of metastasis (Table**** 1). A recent study showed that NDUFB6 is a possible tumor suppressor of metastatic clear cell renal cell carcinomas (Narimatsu et al., 2015). NDUFB6, a component of respiratory chain complex I (RCI), is located in the mitochondria (Loublier et al., 2011) and plays an important role in electron transfer activity (Dieteren et al., 2011). RCI dysfunction may contribute to tumor progression by enhancing the metastatic potential of tumor cells (Narimatsu et al., 2015). Clinical study has indicated that downregulation of NDUFB6 was implicated in poor prognosis of clear cell renal cell carcinomas (Narimatsu et al., 2015).

Invasion and distal metastasis are two important factors affecting the survivor rate of patients with gastric cancer. In this study, we found that NDUFB6 expression was associated with invasion and distal metastasis (Table**** 1). These mean that NDUFB6 may be used as a biomarker of metastasis for patients with gastric cancer. Since NDUFB6 is one member of the metabolic‐associated lncRNAs–mRNAs coexpression network (Figure**** 2), the results from our studies indicate that this network may contribute to gastric cancer development.

## 5 CONCLUSIONS

In summary, our results provide clues about the metabolic functions of lncRNAs in gastric cancer. The metabolism‐associated lncRNAs play important roles in the occurrence and development of gastric cancer. They may be used in the diagnosis of cancers such as gastric cancer.

## DECLARATIONS

### Ethics approval and consent to participate

All aspects of this study were approved by the Human Research Ethics Committee of Ningbo University (IRB No. 20120303). Written informed consent was obtained from all patients.

### Consent for publication

Not applicable.

### Availability of data and material

https://www.ncbi.nlm.nih.gov/geo/query/acc.cgi?acc=GSE96856.

### Competing interests

The authors declare that they have no competing interests.

## AUTHORS’ CONTRIBUTIONS

XM: designing experiment, conducting experiments, acquiring and analyzing data, writing and editing the manuscript; TL: collecting tissue samples, conducting experiments, acquiring and analyzing data; LZ conducting experiments, acquiring and analyzing data; BX: designing experiment, analysis and critical evaluation of the data; QL: designing experiment, conducting experiments, analyzing data, writing and editing the manuscript; JG: concept of the study, designing experiments, providing samples and reagents, writing and editing the manuscript. All authors have read and approved the final manuscript.
